# Myopia control and modulation transfer functions for multisegment spectacle lenses

**DOI:** 10.1111/opo.70016

**Published:** 2025-09-17

**Authors:** W. Neil Charman, David A. Atchison

**Affiliations:** ^1^ School of Health Sciences, Faculty of Biology, Medicine and Health University of Manchester Manchester UK; ^2^ Centre for Vision and Eye Research Queensland University of Technology Brisbane Queensland Australia

**Keywords:** DIMS, modulation transfer function, multisegment, myopia, myopia control, spectacles

## Abstract

**Introduction:**

Multisegment (MS) spectacle lenses, providing a distance correction with a clear central area and by having an array of small, positively powered lenslets in the periphery of the front surface, have proved effective in slowing childhood myopia progression. Debate continues as to whether their mechanism of action is due to through‐focus effects or to the image contrast changes due to the inclusion of the lenslets. This study explores the second possibility by modelling the performance of a combined MS lens‐eye optical system in terms of its modulation transfer function (MTF) under various conditions.

**Methods:**

The optical design program Ansys Zemax OpticsStudio was used to determine distance MTFs for the combination of either a single‐vision or a Hoya MiyoSmart MS lens with a 4 D myopic eye model. Conditions included axial and peripheral objects with co‐axial lens and eye, and rotating the eye away from the lens axis to observe objects through the lenslet‐covered region of the lens. Visual resolution under each condition was estimated.

**Results:**

Observing objects through the lenslet array lowered modulation transfer in comparison with that given by the single‐vision lens, especially as spatial frequency increased. In peripheral observation at a field angle of approximately 32.5 degrees, imagery was poor. Foveal image quality was better with axial viewing through the clear MS lens centre than when the eye was rotated by approximately 30 degrees.

**Conclusions:**

Optimal visual resolution during MS lens wear is achieved when fixating through the clear, central area of the lens. Under these circumstances, objects at 20–50 degrees from fixation are seen through the lenslet‐covered region of the carrier which produces a low‐pass spatial frequency filtering effect. Here, visual resolution is limited to a few cycles per degree so that any growth control mechanism must rely on low spatial frequency information.

**Key points**

- Foveal modulation transfer function and vision are always worse when objects are viewed through the lenslet‐covered areas of multisegment lenses than through their clear central area.
- In both foveal and peripheral vision, the presence of lenslets within an imaging beam primarily reduces modulation transfer at relatively higher spatial frequencies in comparison with that given by the single‐vision carrier lens.
- The poor quality of the optical images and neural performance in the peripheral retina implies that any impact of multisegment lenses on the control of myopia progression involves relatively low spatial frequency information (a few cycles per degree).

## INTRODUCTION

With the rise in myopia prevalence in many parts of the world, interest has grown in developing treatments to reduce the excessive age‐dependent changes in axial length which are primarily responsible for the myopia. One approach has been specially‐designed spectacle lenses.^1^ Many of these are of multisegment (MS) design and are based on single‐vision, or carrier lenses. The front surfaces have small, usually positively powered, refractive elements, e.g., circular lenslets arranged in various patterns or narrow, concentric, cylindrically powered annuli.^1^ The central areas of the lenses are lenslet‐free.

The power of a lenslet is given by the difference in powers between its front surface and the carrier front surface. Ideally, the junction of the back surface of a lenslet and the carrier lens has zero power as the back surface of the former and the front surface of the latter have the same curvature (physically they are continuous). However, the junction of the periphery of the front surface of a nominally positive lenslet and the carrier may effectively form a concave surface, which would give local negative power. Negative power may also result from asphericity of the lenslets if this is such that the radial curvature of the peripheral anterior surface becomes negative in the lenslets' periphery.^2^, ^3^ This effect may be exaggerated if any hard or other coating is applied to the lens.

It has been hypothesised that the additional elements produce, in the peripheral image field, a second ‘myopic’ image surface lying anterior to the retinal image produced by the carrier lens. Animal experiment results have suggested that this arrangement may slow the rate of axial eye growth.^4^, ^5^, ^6^, ^7^, ^8^

Large‐scale, well controlled, wearing trials extending several years have shown that daily wearing of these MS lenses, or of contact lenses based on similar principles, reduces the rate of increase of both axial length and myopia in childhood.^9^, ^10^ Moreover, vision remains satisfactory even if the eye rotates behind the lens to fixate on an object through the lenslet‐covered area, rather than through the central clear area.^11^, ^12^, ^13^

Despite this success, the mechanism of action for myopia control by the lenses remains unclear. Remarkably, several different designs of lenses of this type appear to give similar quantitative myopic control effects, even though the powers and shapes of their segments differ widely. Moreover, a study using lenslets with powers of the same magnitude, but opposite sign, shows that both positively‐ and negatively powered lenslets produce similar myopia‐control effects, suggesting that the ‘myopic defocus’ concept is, at best, incomplete.^14^

The further discovery that other lenses, with surfaces having irregular arrays of scattering elements, also inhibit myopic progression implies that a loss in image contrast may be an important factor in the control effect of MS lenses.^14^, ^15^, ^16^, ^17^

We have recently investigated theoretical models of the retinal imagery of point objects, as seen through eyes wearing one of three designs of MS lens. This work had an emphasis on imagery when the light rays pass obliquely through the lenses and the aberrations of both the lens and the eye affect the results.^2^, ^3^, ^18^ This study now considers the corresponding lens‐plus‐eye modulation transfer functions (MTFs) for one of these lenses, the Hoya MiyoSmart (Defocus Incorporated Multiple Segments (DIMS)) MS‐lens (hoyavision.com/vision‐products/miyosmartVr25), to explore further the possible mechanism of action of MS lenses.

In particular, the following questions are addressed:

(i) What is a typical lens‐eye MTF when the axes of the lens and eye are coincident, so that the eye is looking at a distant object on the axis through the clear central area of an MS lens, and how does this change if the lenslet array is extended to cover the clear area? Several earlier studies of the MiyoSmart lens have considered theoretically an approximation to this situation, with a distant axial object and the simplifying assumptions that the carrier lens is afocal and the aberration‐free eye is emmetropic.^11^, ^19^, ^20^
(ii) If the axes of the eye and carrier lens remain coincident, with no rotation of the eye, how does the MTF for an object at a fixed angle in the distant peripheral field change when it is first viewed peripherally through the single‐vision carrier lens and then through the lenslet‐covered region of a complete MS lens? That is, for a stationary eye, what changes in the peripheral MTF are induced by adding the lenslets to the carrier?
(iii) In relation to the resolution achieved when looking through clear and lenslet‐covered parts of the lens, what is the difference between the MTF when the axes of the lens and eye are coincident so that the eye is looking at a distant object on the axis through the clear central area of the MS lens, and that achieved when the eye rotates behind the MS lens to view a distant object originally at a field angle of about 30 degrees through the lenslet‐covered periphery of the MS lens? Is any reduction in MTF in the second case likely to degrade photopic resolution?

## METHODS

The modelling for imaging point objects by either the lens alone or the lens‐eye combination has been described fully in previous papers.^2^, ^3^, ^11^, ^18^, ^19^, ^20^ Although numerous possible configurations may occur in practice, the basic (and we hope, reasonably typical) model discussed here consists of a Hoya MiyoSmart lens, having lenslets arranged in a concentric series of hexagons,^2^, ^19^ combined with an adult model eye whose parameters vary with refractive error and accommodation.^21^, ^22^ In the model, parameter values were chosen to be representative of real eyes and to simulate their aberrations. Refraction‐dependent parameters of the eye are anterior corneal curvature, vitreous length and the vertex radii of curvature and asphericities of a biconic retina. For the purposes of the present paper, which only concerns imaging distant objects, it was assumed that the spectacle‐plane refraction was −4 D at 12 mm in front of the unaccommodated eye. The stop diameter was taken to be 5.0 mm. With this diameter, the effects of the eye model's under‐corrected spherical aberration^23^ made it necessary to reduce the original model's axial length by 0.12 mm in order to optimise the axial MTF for distance.

The Optical Design program Ansys Zemax OpticStudio R2.02 (ansys.com/products/optics/ansys‐zemax‐opticstudio) was used to evaluate various aspects of the lens‐eye combination. Previously, we reported spot diagrams and fast Fourier transform point‐spread functions^2^, ^3^ and power errors across the pupil and across the field.^3^, ^18^ In the present study, fast Fourier transform optical transfer functions were calculated. The absolute values of the complex optical transfer functions gave modulation transfer functions (MTFs). The output MTF was in terms of cycles per mm (c/mm) in the retinal image. A method to convert c/mm in the image space to cycles per degree (c/degree) in the object space is described in Appendix 1.

Three imaging conditions were considered. In the first, the optical axes of the carrier lens and eye coincided and the MTF was evaluated for a distant, axial object. The light beam passed through the lenslet‐free central area of the MS lens and image degradation was due only to the combined effect of the axial aberrations of the carrier lens and eye. A variant of this model added four additional hexagons of lenslets to the centre of the carrier lens,^2^ to simulate the case considered by earlier authors in which the chief ray is incident normally on the lenslets.^11^, ^19^, ^20^, ^24^

The second condition represented peripheral vision. The optical axes of the lens and eye remained coincident, but the distant object was at a vertical peripheral field angle such that the chief ray passed through the centre of a lenslet in the third hexagon from the centre when the longest diameters of successive hexagons were aligned vertically, corresponding to an object field angle of 32.5 degrees. The MTF was calculated for both the MS lens and for the carrier lens only.

Finally, in the third condition, rather than the lens and eye axes remaining coincident, the eye was rotated 31.7 degrees about its centre of rotation so that a distant object at a vertical object field angle of 35.8 degrees could be viewed foveally; the difference between the two angles being due to the prismatic effect of the −4 D carrier lens. The chief ray passed through the centre of a lenslet in the eighth hexagon from the centre. Again, the MTF was calculated for both the MS lens and for the carrier lens only.

## RESULTS

**FIGURE 1:**
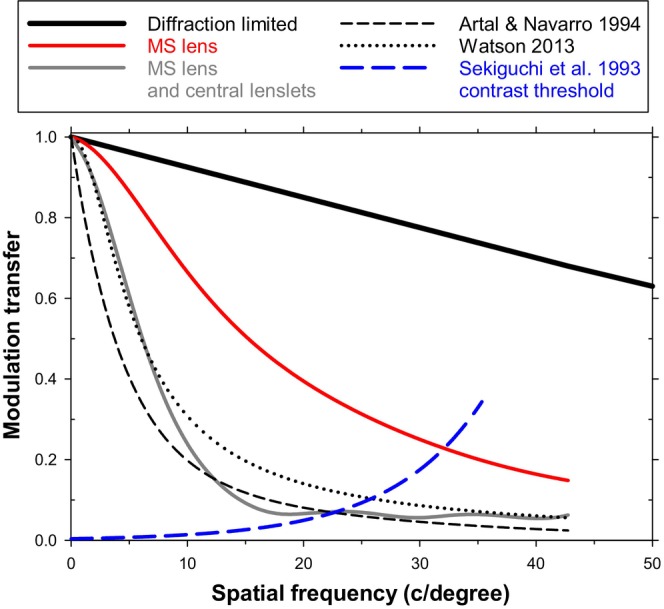
Axial modulation transfer functions (MTFs) for combined multisegment (MS) lens‐unaccommodated eye system with 5 mm stop diameter: Diffraction‐limited system (thick black line), standard MS lens with lenslet‐free central aperture (red line), MS lens and added simulated central lenslets (grey line), average double‐pass based experimental result of Artal and Navarro^25^ (short dashed black line), and average wavefront‐error based experimental result of Watson^26^ (dotted black line). Also shown are foveal modulation thresholds (Sekiguchi et al.^27^) for sinusoidal gratings (dashed blue line).

Figure 1 shows the axial MTFs for a diffraction‐limited eye, for the MS lens with a clear central area which acts axially like a single‐vision lens and for the MS lens with simulated central lenslets, that is, no clear area. Typical mean experimental results for young adult eyes with a 5 mm entrance pupil, as obtained by double‐pass (Artal and Navarro^25^) and wavefront‐error (Watson ^26^) techniques, are included for comparison. Both the model's predictions and the experimental measurements show that, within the spatial frequency band which is resolved in normal human vision, the in‐focus MTF for a 5 mm pupil falls steadily with spatial frequency. Adding lenslets to the correcting carrier lens lowers the MTF at all spatial frequencies.

Figure 1 also shows Sekiguchi's neural contrast thresholds for sinusoidal gratings under photopic conditions.^27^ For an observer to be able to resolve the retinal image of a grating with a modulation of one (i.e., maximal contrast), the modulation transfer must be higher than the contrast threshold. The resolution for a high‐contrast sinusoidal gratings is found at the intersection of an MTF with the neural threshold plot. This is considered further in the Discussion.

**FIGURE 2:**
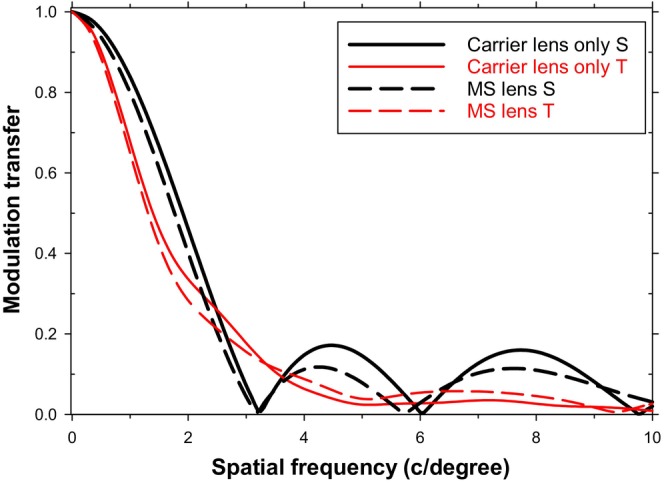
Modulation transfer functions (MTFs) in peripheral vision with a stationary eye. Field angle is 32.5 degrees. Single‐vision, lenslet‐free carrier lens sagittal (S) (solid black line), carrier lens tangential (T) (solid red line), full MS lens with peripheral lenslets S (dashed black line), multisegment (MS) lens T (dashed red line). There are phase reversals at about 3 and 6 c/degree for the two sagittal MTFs. Note change in horizontal scale from that in Figures 1 and 3.

Figure 2 shows MTFs when the optical axes of the eye and MS lens remain coincident, but the distant object is viewed in peripheral vision at a vertical object field angle of 32.5 degrees, either through the single‐vision carrier lens alone or through the lenslets of the full MS lens. The sagittal case (S) corresponds to gratings with vertical bars, and the tangential case (T) corresponds to gratings with horizontal bars. Image quality is better for the carrier lens than for the MS lens, whose lenslets degrade MTFs. In both cases, there is a steep initial fall in modulation transfer with spatial frequency and a substantial difference between the sagittal and tangential MTFs due to oblique astigmatism. There are reversals of phase (spurious resolution) at spatial frequencies above 3 c/degrees.

**FIGURE 3:**
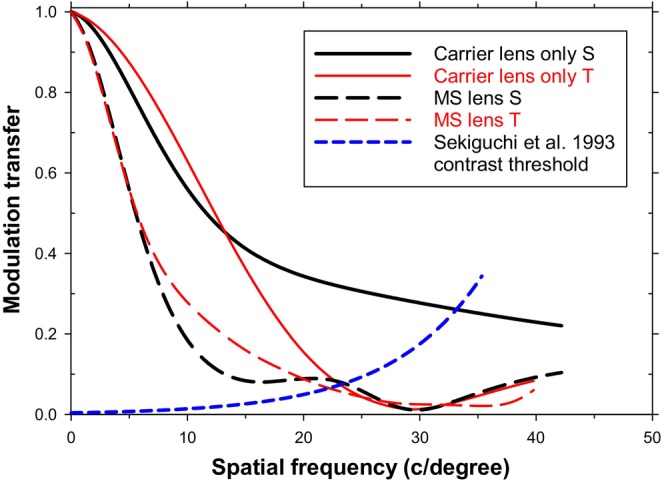
Modulation transfer functions (MTFs) for rotating eye and foveal vision. Eye rotation angle 31.7 degrees (object field angle 35.8 degrees) through the 8th lenslet from the centre. Since there are only nine hexagons of lenslets and the stop is 5 mm in diameter, the full imaging beam just overlaps the outer edge of the lenslet array. Carrier lens only sagittal (S) (solid black line), carrier lens only tangential (T) (solid red line), multisegment (MS) lens S (dashed black line), MS lens T (dashed red line). Also shown are foveal modulation thresholds (Sekiguchi et al.^27^) for sinusoidal gratings (dashed blue line).

Figure 3 shows MTFs when the eye rotates through 31.7 degrees to view foveally an object at a field angle of 35.6 degrees. The MTFs are plotted again for the cases of the carrier lens and the MS lens. The MTFs are better than those for their off‐axis peripheral counterparts (Figure 2). The carrier viewing condition shows slight oblique astigmatism attributable to the obliquity of the light beams through the lens. The addition of the lenslets of the MS lens has major effects on both the sagittal and tangential MTFs.

## DISCUSSION

When interpreting these results, it must be borne in mind that the parameters of the lens‐eye model used were appropriate to adult, rather than children's eyes and that the object configurations studied represent only a very small sample of the possible viewing conditions, e.g., degree of myopia, pupil diameter, object field angle, object distance, ocular rotation, position of chief ray with respect to lenslets, etc. The individual variations in ocular aberrations were not considered, nor the Stiles‐Crawford effect which, if included, would have improved imaging performance slightly.^28^ Ocular and spectacle lens aberrations, etc., will vary with the degree of myopia, and the instrumentation used to determine the shape of the lenslets in our lens model^19^ may not be capable of recording any small blending zones between the lenslet and carrier surfaces. Restriction of the calculations to monochromatic conditions is also a limitation. Nevertheless, we believe that these and other deficiencies do not have major effects on the general trends found in the current results and that the present calculations do give valuable insights into the possible mechanism of action of the lenses in myopia control. Note that these results refer to the image plane for the carrier lens rather than that for the lenslets or elsewhere. This is reasonable, since previous studies have shown that, in tracking accommodation stimuli, wearers always seek to focus on the carrier's image plane,^29^ rather than that of the lenslets which are superimposed on the carrier. This is almost certainly because the in‐focus images formed by the individual lenslets are laterally displaced with respect to one another, making the composite ‘lenslet’ image a poor accommodation stimulus (e.g., Radhakrishnan et al.^24^).

### Axial objects, coincident lens and eye axes

Considering first the axial cases in Figure 1, the axial aberrations of the lens and eye (mainly spherical aberration of the eye) reduce the MTF markedly in comparison with the diffraction‐limited case. Introduction of central lenslets further reduces the MTF of the MS lens at all spatial frequencies in comparison with that when the central area is lenslet‐free. The axial MTF with central lenslets is generally similar to earlier published results for lenslet‐covered areas of the Hoya MiyoSmart lens,^11^, ^19^, ^20^, ^28^ except that in this result, modulation transfer is generally lower due to the inclusion of the effects of the eye's aberrations.

As would be hoped from the parameters chosen for the model eye, the axial MTF for the single‐vision carrier lens case approximates those found by direct experimental measurement in the young adult eye,^25^, ^26^, ^30^ although the model result is somewhat higher. In the case of the double‐pass results, this may be because the latter include the degrading effects of intraocular scattered light, which are not considered in the model. In general, the model predicts the optical performance of the eye with reasonable success.

The visual performance associated with these optical MTFs will vary with the retinal (or neural) performance, which can be determined as the contrast required for monochromatic, sinusoidal gratings to be resolved. Sekiguchi et al.^27^ gave relevant foveal thresholds for young adult observers at a wavelength of 515 nm and a photopic retinal illuminance of 500 trolands. The wavelength is sufficiently close to our modelling value of 550 nm for it to be possible to deduce the expected foveal resolution limit, or cut‐off frequency for high‐contrast gratings, from the intersection of the MTFs with the threshold curve (Figure 1), although in practice the grating thresholds may vary somewhat with the individual observer, the orientation and colour of the grating and the meridian under study. Figure 1 suggests that the grating resolution limit is 32 c/degree with the MS lens (intersection of solid black MTF plot and dotted blue contrast threshold plot). This compares with a resolution of 22 c/degree for the MS lens with added simulated central lenslets (intersection of solid grey plot and blue plot). Taking logarithms of ratios of resolution shows that the presence of the lenslets is expected to reduce the resolution limit by about 0.15 log units, roughly equivalent to 1.5 lines of a logMAR visual acuity chart. This is not very different from the reductions in visual acuity of about 0.1 log units found experimentally when Landolt C targets were observed by children through the lenslet‐covered area of MiyoSmart lenses as compared with single‐vision lenses.^11^ Not surprisingly, an experimental study has confirmed that, on‐axis, where the MiyoSmart lens is clear, high‐contrast Landolt‐C visual acuity and contrast sensitivity are the same when wearing either MiyoSmart or single‐vision lenses.^31^

**FIGURE 4:**
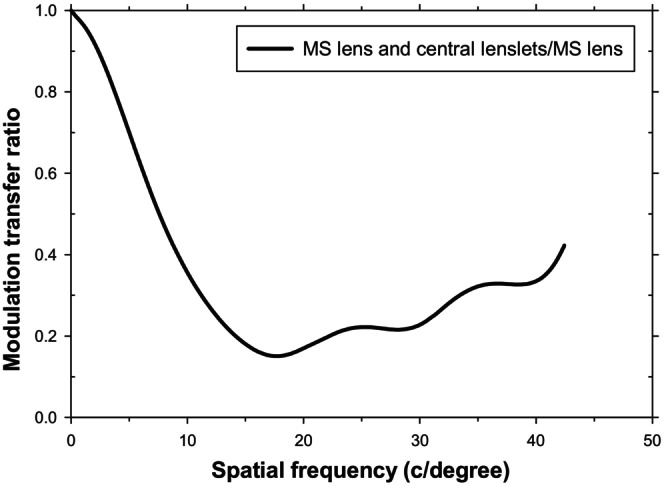
Impact of simulated central lenslets on the axial modulation transfer function (MTF). The modulation transfer of the multisegment (MS) lens with simulated central lenslets, divided by the modulation transfer for the MS lens, is plotted against spatial frequency. Introduction of central lenslets reduces the spatial frequency at which high‐contrast sinusoidal gratings can be resolved from 32 to 22 c/degree.

While the cut‐off frequencies for the detection of high‐contrast sinusoidal gratings are of interest, the related question of the impact of the lenslets on supra‐threshold grating images is also important. The added lenslets can be considered as being equivalent to a filter which reduces the modulation transfer at each spatial frequency. Using the data in Figure 1, Figure 4 shows the nature of this filter. It presents the modulation transfer of the MS lens with added central lenslets divided by that of the normal MS lens with a lenslet‐free central area, which for the axial case acts like the single‐vision carrier lens. The introduction of the lenslets affects vision over the whole useful band of frequencies below the cut‐off value. The filtering is relatively small at lower spatial frequencies (<about 5 c/degree) but becomes more important at higher spatial frequencies; that is, the lenses act as a low‐pass spatial frequency filter.

### Objects in peripheral field (32.5 degrees), stationary eye (i.e., coincident lens and eye axis), peripheral viewing

Figure 2 shows that retinal image quality is very much reduced during peripheral viewing. For both the single‐vision carrier and the MS lens, there is a marked difference between the sagittal and tangential MTFs because of considerable oblique astigmatism in both cases.^18^ All the MTFs fall rapidly with spatial frequency to reach zero at spatial frequencies of about 3.1 and 10 c/degree, respectively, for the sagittal and tangential cases for the single‐vision carrier, and at 3.1 and 9.3 c/degree, respectively, for the corresponding MS lens MTFs. At higher spatial frequencies, modulation transfer values are low. After the modulation transfer reaches zero, there is a phase shift of 180 degrees, that is, half a cycle phase shift (spurious resolution). However, Frisen and Glansholm's work,^32^ using high‐contrast interference fringes to bypass the optics of the eye, indicates that the neural resolution limit for high‐contrast, sinusoidal gratings at these field angles is about 2.3 c/degree, similar to the 3.0 c/degree determined by Thibos et al.^33^ and Zhu et al.,^34^ who found temporal values at a 30 degree field (about 3 c/deg) were higher than nasal values (about 2 c/deg). Thus, the higher spatial frequencies where there are reversals of spatial phase of the image are likely to be unresolvable, although they may be detectable, since peripheral detection thresholds are lower than resolution thresholds.^32^, ^33^

**FIGURE 5:**
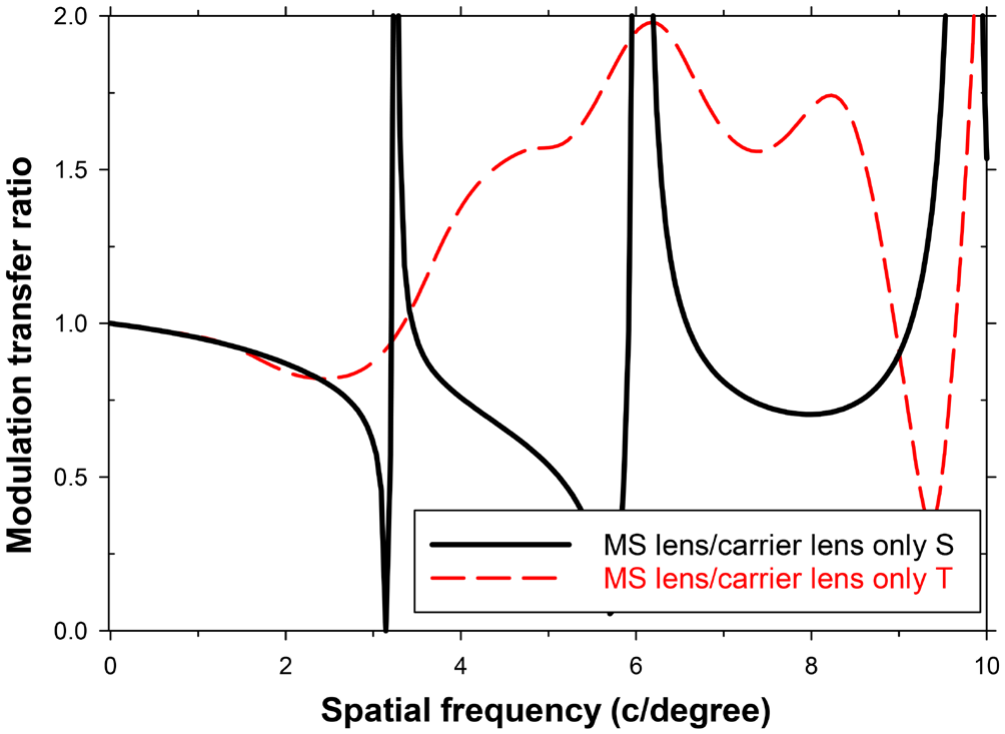
Impact of lenslets on sagittal (S) and tangential (T) modulation transfer in peripheral vision for an object‐space field angle of 32.5 degrees. The modulation transfer of the multisegment (MS) lens, divided by the modulation transfer for the carrier lens only, is plotted against spatial frequency. Note changes in horizontal scale from those in Figures 4 and 8.

Interestingly, for the situation described above, the spatial frequency filtering effects appear to be relatively small. Using the data in Figure 2, Figure 5 shows the MTF ratios obtained by dividing the MTF of the full MS lens by that of the carrier lens. If we assume that spatial frequencies above 3 c/degree play no role in form vision, since they are not resolved, the important feature is that the lenslets cause a degradation in modulation transfer which increases with spatial frequency in both the tangential (T) and sagittal (S) cases, so that the T modulation transfer is reduced by a factor of about 0.6 and the S modulation transfer falls to zero at about 3 c/degree.

Thus far, peripheral vision has been considered at only a single object‐space field angle (32.5 degrees). The implication of these results is that, with these MS lenses, any mechanism which affects eye growth and depends on peripheral imagery through the lenslets must operate either at low spatial frequencies (<3 c/degree in the case studied) and involve relatively small lenslet‐induced changes in modulation transfer or, alternatively, must involve the absence of medium and high spatial frequency information.

**FIGURE 6:**
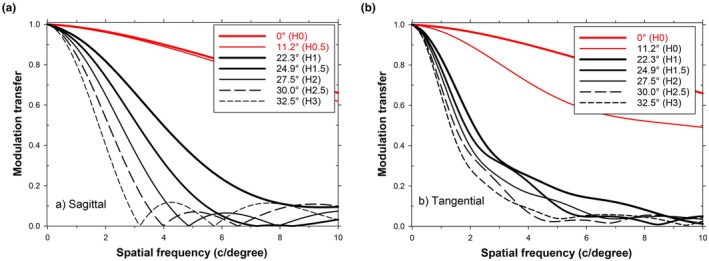
Effect on modulation transfer function (MTF) of changing object field angle in peripheral vision. (a) Sagittal MTFs, (b) Tangential MTFs. Red curves labelled hexagon (H)0 and H0.5 refer to fields for which chief rays pass through the lenslet‐free central area of the lens, and black curves labelled H1.0 to H3.0 refer to field angles for which chief rays pass either through the centre of a lenslet or midway between two lenslets.

It is reasonable to ask whether poor modulation transfer always occurs at medium and high spatial frequencies when peripheral vision involves imaging beams which pass through the lenslets. Figure 6 shows a sequence of peripheral MTFs obtained when fixation is axial. Vertical field positions are such that the chief ray gradually moves across the border between the central clear and outer lenslet‐covered areas of the MS lens. The chief ray passes through either the clear central area, the centre of a lenslet or a clear area halfway between lenslets. For the Hexagon (H)0 and H0.5 plots, the chief ray is either axial (H0) or passes through the clear area of the lens midway between the axis and the first lenslet ring (H0.5). For H1, H2 and H3, the chief ray passes through the centres of lenslets in the first, second and third hexagons, respectively, and in H1.5 and H2.5 the chief ray is incident midway between neighbouring lenslets. The MTFs steadily deteriorate as the field angle increases, due to increasing off‐axis aberrations and to an increasing fraction of the entrance pupil being affected by the lenslet array. The deterioration is greater for the sagittal MTFs (Figure 6a) than for the tangential MTFs (Figure 6b), with the first zero of the former moving to progressively lower spatial frequencies as the field angle increases. For both sagittal and tangential plots, modulation transfer at higher spatial frequencies is always low when mid‐peripheral imaging beams pass through the lenslet array.

**FIGURE 7:**
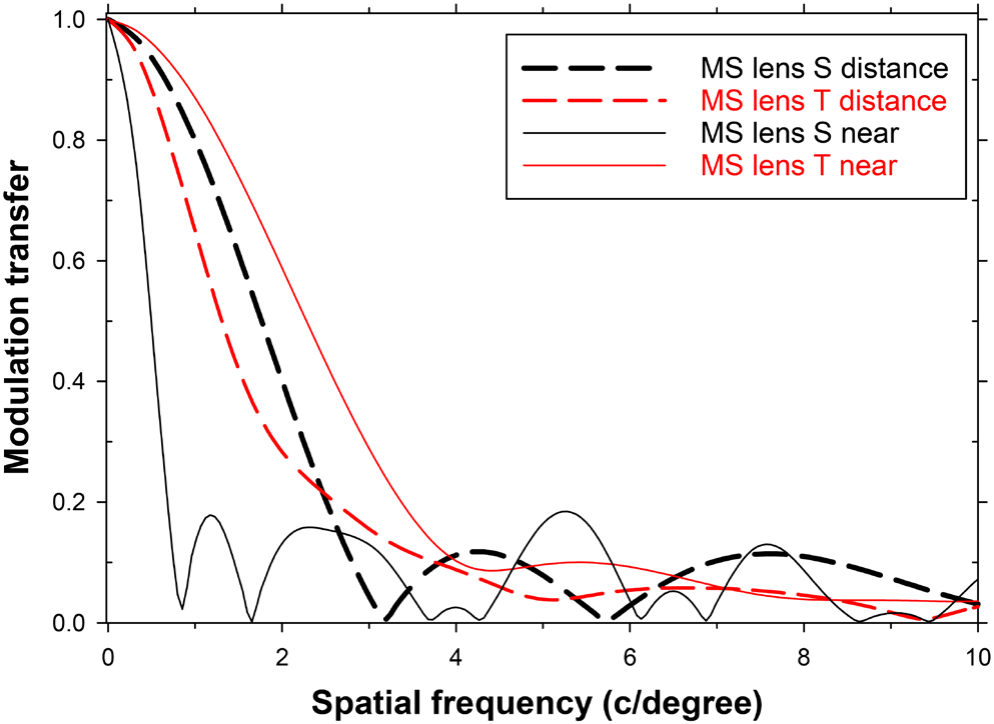
Sagittal and tangential modulation transfer functions (MTFs) for peripheral viewing of an object at a field angle of 32.5 degrees at infinity (zero vergence) and at a finite distance (250 mm, vergence −4 D). MS, multisegment lens; S, sagittal; T, tangential.

One further question of interest is whether, in this peripheral viewing situation with fixation on a distant axial object, the distance of any peripheral object has major effects on its retinal image? Such a situation might arise in many practical tasks where a distant object is directly observed in an environment that also contains proximal peripheral objects. For example, a child might observe a distant blackboard when sitting at a desk covered with books and writing equipment. Accommodation is normally governed mainly by blur in the foveal image, although some exceptions may occur.^35^ If accommodation is foveally stimulated, there will be a shift in the peripheral focus compared with that for the peripheral MTFs of Figures 2 and 6. To illustrate the likely magnitudes of the involved changes in MTF, Figure 7 shows the sagittal and tangential peripheral MTFs for a field angle of 32.5 degrees for two peripheral object distances (infinity and 250 mm), with the eye remaining unaccommodated in both cases. Various detailed differences occur, but the MTFs are such as to suggest that there is a continued absence of medium and high spatial frequency information in the retinal image. In an individual lens‐wearing patient, the exact effects will depend primarily on the relative peripheral refractive errors of the individual eye, although the role of the latter in myopia development remains controversial.^36^, ^37^

### Object viewed foveally with a rotated eye

Figure 3 shows that, in comparison with a single‐vision lens, the MS lens lowers modulation transfer markedly at most spatial frequencies. MTFs for both lenses show the effects of oblique astigmatism. Since vision with the rotated eye is foveal, we can again estimate the expected grating cut‐off frequencies by finding the intersection points between the MTFs and the grating threshold curve.^27^ This gives resolution limits for the sagittal and tangential cases, respectively, of 33 and 22 c/degree for the single‐vision carrier and 22 and 20 c/degree for the full MS lens (intersections of black and red MTF plots with the dotted blue contrast threshold plot). Taking logarithms of ratios of resolution shows that the presence of the lenslets reduces the sagittal and tangential grating resolution by about 0.18 and 0.04 log units, respectively. The combination of aberrations is such that for the single‐vision carrier case, the sagittal resolution with the rotated eye is slightly better than in the non‐rotated axial case (Figure 1). The lens is close to the best shape it can be for the rotating eye.

**FIGURE 8:**
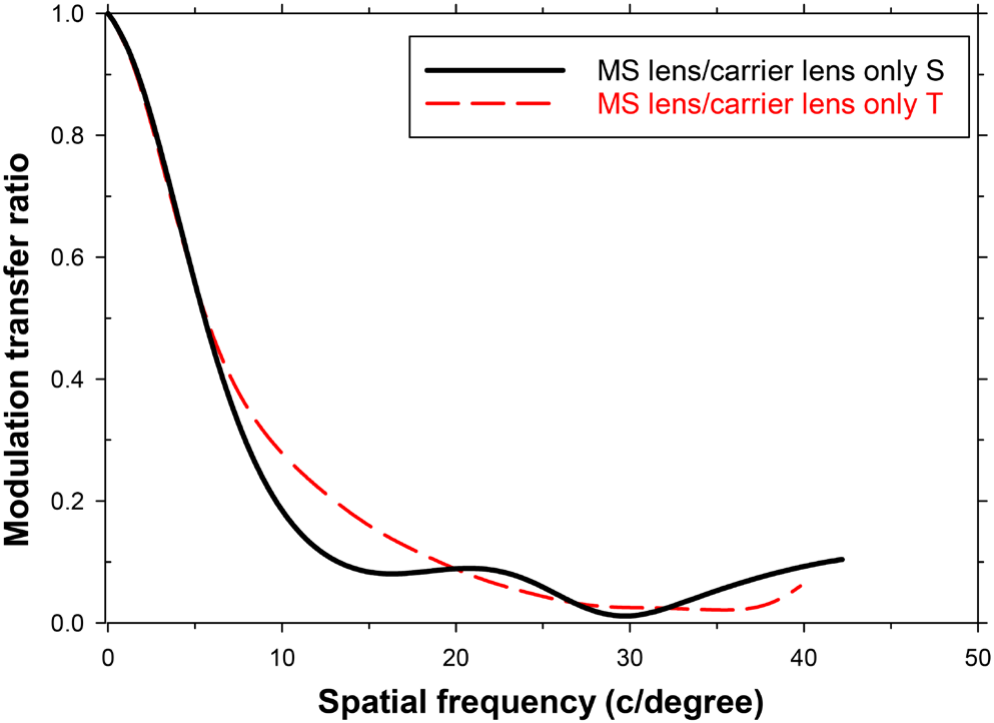
Impact of lenslets on sagittal (S) and tangential (T) modulation transfer for foveal vision and a rotation angle of 32.5 degrees. The modulation transfer of the multisegment (MS) lens, divided by the modulation transfer for the carrier lens only, is plotted against spatial frequency. The peak at 31 c/degree in the tangential case occurs beyond the visual resolution limit.

As in Figures 4 and 5, the effect of the lenslets can be represented in terms of a filter. As shown in Figure 8, the ‘filter’ transmittance decreases from unity at very low frequencies and falls steadily as the spatial frequency increases towards the MS lens resolution limits of 22 and 20 c/degree for the sagittally‐oriented (vertical‐bar) and tangentially‐oriented (horizontal‐bar) gratings. The behaviour above these limits is irrelevant because these spatial frequencies do not contribute usefully to form vision. Once more, then, the lenslet array is acting as a low‐pass spatial frequency filter.

**FIGURE 9:**
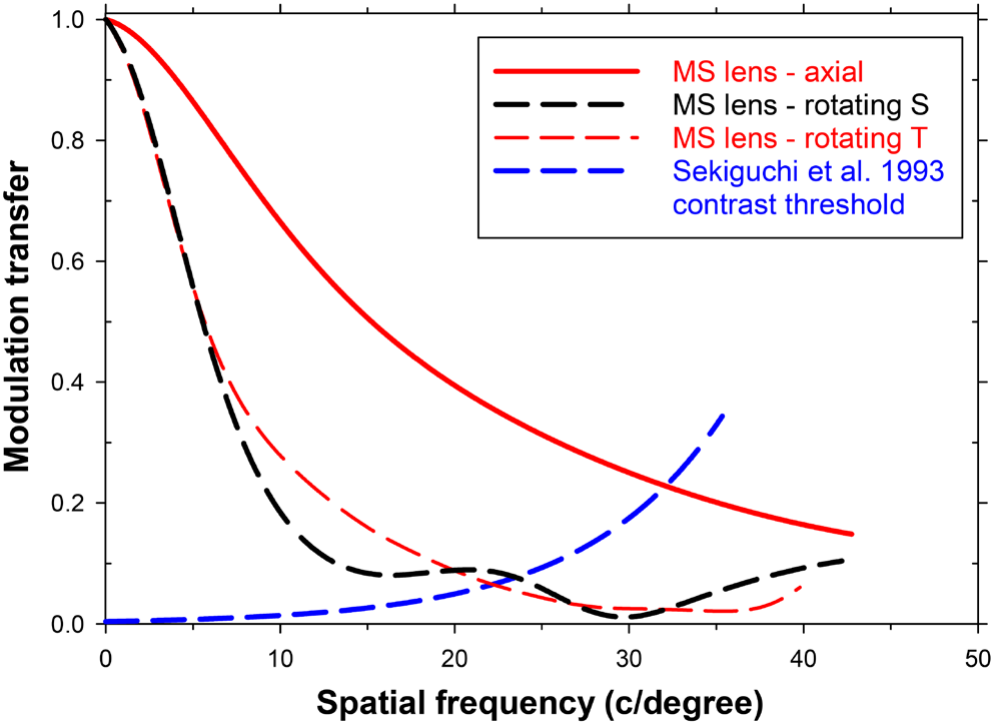
Modulation Transfer Functions (MTFs) for an eye looking through lens centre (axial) and for a rotating eye (31.7 degrees) with foveal vision. Multisegment (MS) lens axial (solid red line), MS lens sagittal (S) (dashed black line), MS lens tangential (T) (dashed red line). Also shown are foveal modulation thresholds (Sekiguchi et al.^27^) for sinusoidal gratings (blue dashed line).

The chief interest in this case, however, is in the information it gives on how patients should use their MS spectacles. In any practical situation, the wearer of an MS lens normally has the choice of either viewing an object directly, through the clear central area, by means of a head turn or of keeping the head steady and rotating the eye to obtain foveal fixation, with the consequence that the object is viewed through the lenslet‐covered area. Obviously a third choice is to use some combination of these movements. Figure 9 compares the MTFs appropriate to these two conditions (from Figures 1 and 3) with the Sekiguchi et al.^27^ retinal threshold. As can be seen, the sagittal and tangential MTFs for the 31.7 degree eye rotation are markedly lower than the axial MTF with no eye rotation. The estimated resolution limit for sinusoidal gratings is 32 c/degree in the axial case, whereas the sagittal and tangential MTFs appropriate to viewing through the lenslets in the rotated eye situation give visual cut‐offs of about 22 and 20 c/degree, respectively, with the astigmatism being due to the oblique viewing through the spectacle lens. This suggests that changing from head‐turn to eye‐turn viewing might cause a fall in grating resolution of about 0.16 and 0.20 log units in the sagittal and tangential cases, respectively. This compares with experimental findings of logMAR visual acuity losses of about 0.05 to 0.1 log units when the eye rotation is about 20 degrees.^11^, ^12^, ^13^ A further comparison between the peripheral conditions and the rotating eye concerning the effect of the lenslets is given in Appendix 2.

**FIGURE 10:**
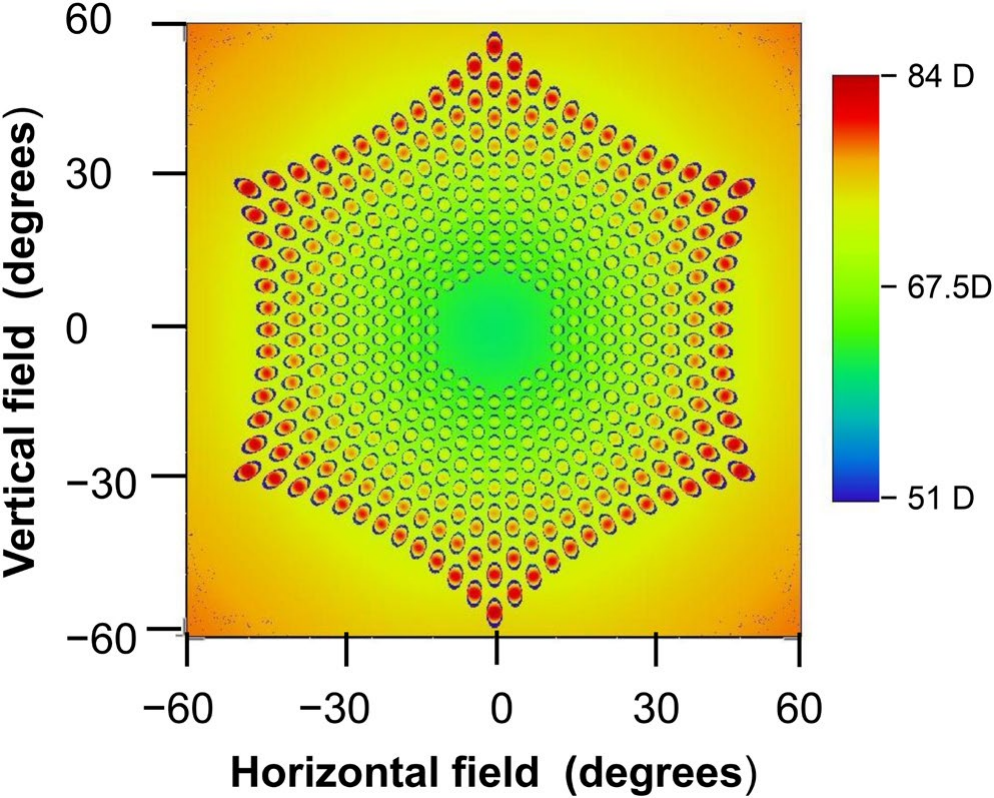
Tangential power plot across the visual field when fixation is axial. The object field seen through the lenslets lies roughly between 20 and 50 degrees but with hexagonal boundaries.

It may therefore be advantageous to maintain fixation through the clear lens centre by turning the head to view objects rather than by rotating the eyes. Under these circumstances, the pupil can receive light rays which have passed through a lenslet in passage from a hexagonal annular area of the peripheral object field, centred on the common carrier lens/eye axis. Assuming that the longest diameter in the hexagonal annulus of lenslets is vertical and that limiting cases occur when a lenslet from either the innermost or outermost hexagon of lenslets appears central in the entrance pupil, the corresponding smallest inner and outer semi‐diameters of this hexagonal area of object field are about 22 and 58 degrees, respectively, in the vertical meridian and 20 and 52 degrees in the horizontal meridian. This situation is illustrated in Figure 10 which shows a tangential power field plot, with powers referenced to the second principal plane after the last refracting surface of the eye. Figure 10 can be interpreted as giving, for any point in the object field when fixation is on the common lens/eye axis, the location of the chief ray as it passes through the lenslet array of the complete MS lens. If no eye rotation occurs, any direct effect of the lenslets would be confined to the corresponding area of the mid‐peripheral retina.

Any myopia‐control effect of the MS lens must therefore arise from neural signals originating in this retinal area. Any rotation of the eye away from the lens axis will, of course, mean that the intersections with the retina of the ray beams which have passed through the lenslets are correspondingly displaced. Such effects are absent with contact lens variants of MS designs, since the lens moves with the eye.

An obvious question is whether the geometry of the lenslet‐covered area of the current MiyoSmart or other MS lenses is optimal for myopia control. Would smaller or larger areas of lenslet arrays produce greater reductions in the rates of change of axial length and refractive error? No serious work has yet been done to answer this question. In the peripheral vision case considered in Figure 2, the object field angle is 32.5 degrees, and the ray beams pass through a lenslet lying near the centre of the width of the field zone affected by the lenslets (about 20–56 degrees). As shown earlier, in this case, photopic high‐contrast grating resolution is only possible for spatial frequencies up to around 3 c/degree. It might be expected that at such low spatial frequencies, the visual system would be relatively insensitive to changes in focus caused by eye growth. In contrast, ray beams that pass through lenslets positioned closer to the lens axis would be incident on more central areas of the retina, where resolution and sensitivity to focus changes are greater.^38^, ^39^ They might, therefore, be likely to have a greater influence on refractive development. This could suggest that the adoption of a smaller inner diameter for the hexagonal area of lenslets could give better myopia control.

In general, the results support the view that, for viewing situations in which light beams pass through their lenslets, MS lenses cause a loss in image contrast in comparison with that for single‐vision lenses. For such imaging beams, the addition of the lenslets makes the MS lenses act like a low‐pass spatial frequency filter and causes a greater loss of information at higher spatial frequencies.

## CONCLUSIONS

If fixation is maintained relatively close to the lens axis, the lenslets of MS Hoya MiyoSmart design primarily affect images falling on the mid‐peripheral retina (corresponding to field angles of about 20–55 degrees). Imagery of axial objects through the clear central area of the lens is the same as that achieved with a single‐vision lens. For objects viewed peripherally at larger, lenslet‐affected field angles, the lenslets cause a progressive reduction in modulation transfer as the spatial frequency rises. Peripheral images are of poor quality. This would be expected to result in a loss in peripheral visual acuity and contrast sensitivity. Any myopia‐control signals generated by the periphery must be dependent on a mechanism which makes use of information at quite low spatial frequencies (a few c/degree).

If the eye is rotated to view foveally an initially‐peripheral object through the lenslet‐covered area of the lens, modulation transfer is much improved in comparison with the non‐rotating eye case where the image falls on the peripheral retina, but the effects of the lenslets cause modulation transfer at medium and high spatial frequencies to be worse than that achievable with a single‐vision lens. Optimal MTF is always obtained by turning the head to view the object foveally through the centre of the lens.

We note that all our modelling relied on lens data available from the manufacturer and the limited number of independent studies of the form of the lenses.^2^, ^3^, ^18^, ^19^, ^20^, ^24^, ^40^ Future work may reveal that current lenses have characteristics which differ slightly from those assumed, particularly in the shape and power of the lenslets. We do not believe that these would be likely to affect the general nature of these results. The loss of image modulation transfer and contrast caused by the lenslets could play a role in the ability of MS spectacle lenses to slow the rate of change in axial length and refractive error in childhood.^15^, ^17^, ^41^, ^42^

## AUTHOR CONTRIBUTIONS

**W. Neil Charman:** Conceptualization (lead); formal analysis (lead); writing – original draft (equal). **David A. Atchison:** Methodology (lead); writing – original draft (equal).

## FUNDING INFORMATION

None.

## CONFLICT OF INTEREST STATEMENT

The authors declare no conflicts of interest.

## Figures and Tables

**FIGURE A1:**
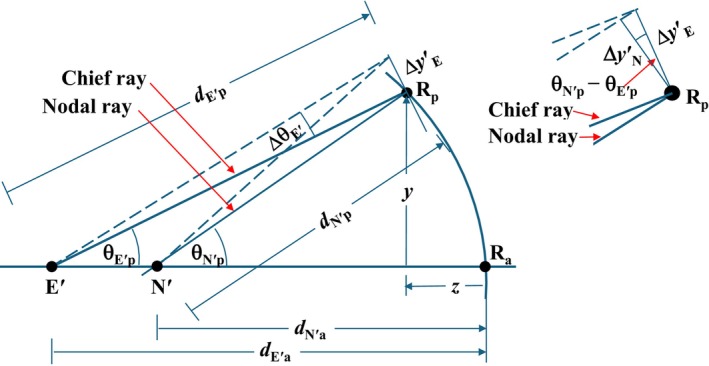
Parameters for determining conversion from c/mm to c/degree for peripheral vision.

**FIGURE A2:**
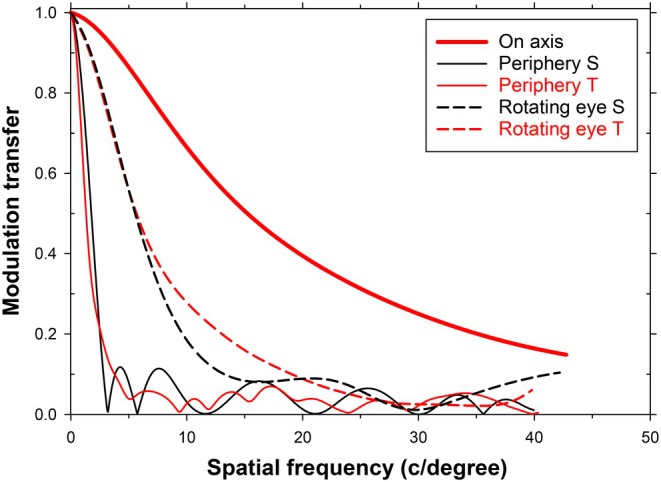
Modulation Transfer Functions (MTFs) for axial, peripheral and rotating eye conditions. S, sagittal; T, tangential.

## APPENDIX 1 Conversion from cycles/distance to cycles/angle

The spatial frequency in c/mm on the retina, given in planes containing and perpendicular to the chief ray, was converted to c/degree, referenced to the second nodal point of the lens‐eye combination. This was done to give angular spatial frequencies applicable to object space. Raytracing was done through the carrier lens in case of minor deviations between carrier only and the full lens.

### Co‐axial lens and eye – axial (foveal) and peripheral vision

The data provided directly by the raytracing program were: object field angle θ_N′p_, axial distance *d*_E′a_ from the exit pupil E′ to the retina at *R*_a_, the peripheral distance *d*_E′p_ to the retina at *R*_p_, the distance from the (paraxial) nodal point to the retina $d_{N\prime a\left(\text{parax}\right)}$, the angle θ_E′p_ of the peripheral chief ray relative to the axis, and the height *y* and sag *z* of the chief ray at *R*_p_ (Figure A1).

The Fast Fourier Transform modulation transfer function was specified in c/mm in planes perpendicular to the chief ray and passing through *R*_p_. Note that these planes did not correspond to the retinal surface, since for peripheral images the chief ray may not be normal to the retina.

Considering the radial plane (in the plane of the paper, Figure A1 left), for a distance Δ*y*′_E_ on this plane corresponding to a cycle, the corresponding angle Δθ_Ε′_ subtended at E′ was

(A.1)
$${\Delta \theta }_{E^{\prime }}/∆y\prime_{E}=1/d_{E\prime p}$$

that is,

(A.1b)
$${\Delta \theta }_{E^{\prime }}=∆y\prime_{E}//d_{E\prime p}$$

Corresponding linear and angular spatial frequencies were 1/Δ*y*′_E_ and 1/Δθ_Ε′_, so the conversion factor from cycles/distance to cycles/angle was $d_{E\prime p}$ with unit mm/radian. A conversion from c/radian to c/degree required a further factor of π/180, so the full conversion at E′ was

(A.2)
$$d_{E\prime p}\;\pi /180$$

Rather than use the second nodal point, which is a paraxial location, a ‘finite’ nodal point was used, which subtended an angle between the axis and the peripheral retinal point that was the same as the object field angle. For simplicity, this is referred to as the nodal point.

From the retinal intersection (*y, z*) and the nodal ray angle θ_N′p_, the distance $d_{N\prime p}$ from the nodal point to the retinal intersection was obtained as

(A.3)
$$d_{N\prime p}=\frac{y}{\sin\left(\theta_{N^{\prime }p}\right)}$$

The corresponding axial distance was

(A.4)
$$d_{N\prime a}=\frac{y}{\tan\left(\theta_{N^{\prime }p}\right)}-z$$

Note that this could be applied to the axial case for which we simply needed the paraxial nodal point distance to the retina, here called $d_{N\prime a\left(\text{parax}\right)}$.

Values in the study for the third hexagon were θ_N′*p*_ = 32.49 degrees, $d_{N\prime a\left(\text{parax}\right)}$ = 16.2101 mm, *d*_E′a_ = 20.8894 mm, *d*_E′*p*_ = 19.9998, θ_E′*p*_ = 24.7205 degrees, and (*y*, *z*) = (8.1933, −3.0749) mm. The distances $d_{N\prime a}$ and $d_{N\prime p}$ were calculated from Equations (A.4) and (A.3), respectively, as 15.9647 and 15.2644 mm, respectively. The distances $d_{N\prime a\left(\text{parax}\right)}$ and $d_{N\prime p}$ replaced $d_{E\prime p}$ in Relation (A.2) to give conversions from c/distance on the retina to c/degree in object space for axial and peripheral vision, of 0.2829 and 0.2664, respectively.

For peripheral vision, there was an additional correction factor in the radial plane (Figure A1 right), where Δ*y*′_E_ projected onto the normal to the nodal ray as Δ*y*′_N_ = Δ*y*′cos(θ_N′p_ − θ_E′p_). This had a minifying effect, increasing spatial frequency by a factor of 1/cos(θ_N′p_ − θ_E′p_). The difference between the chief ray and ‘nodal’ ray in image space was an angle of 32.4900–24.7211 = 7.7689 degrees. The factor was 1.0091. This gave the conversion as 0.2644 × 1.0091 = 0.2689.

The above describes the conversion in the radial plane that will affect the tangential MTF. Considering the conversion orthogonal to the radial plane, which we refer to as the horizontal magnification, the only differences between the exit pupil point and second nodal point cases were their distances from *R*_p,_ that is, the conversion for the sagittal MTF in the peripheral field was 0.2664.

### Central (foveal) vision with rotating eye

The procedure given in the previous section will not work for this case where the chief ray is along the optical axis of the eye. Fields were traced for which the chief ray in image space was displaced ±0.1 mm vertically (*y*′) or 0.1 mm horizontally (*x*′) from the chief ray for the 31.65 degrees rotation condition (35.76 degrees object angle) (Table A1). Note that these angles are rounded to 31.7 and 35.8 degrees in the text, but here greater precision is required. The corresponding object field angles were θ_*y*_ and θ_*x*_. Conversions from c/mm in image space to object field angle were |Δ*y*′/Δθ_*y*_| and |Δ*x′*/Δθ_*x*_|, where the delta sign indicates differences between chief rays. The results for positive and negative values of *y*′ were averaged to give conversions of 0.2653 and 0.2813 along the vertical (radial) and horizontal directions, respectively.

**TABLE A1:** Cycle/degree determinations for central vision with rotating eye.

Chief ray co‐ordinates in plane of retina			Object field direction cosines *l*, *m*, *n* in *x*, *y* and z directions, respectively			Object field components		Conversion
*x*′	*y*′	*z*′	*l*	*m*	*n*	θ_*x*_	θ_*y*_	
0	0	0	0	−0.58442353	0.81144879	0	35.7622757	
0	0.1	0	0	−0.57907600	0.81527357	0	35.3855793	0.2655
0	−0.1	0	0	−0.58975394	0.80758300	0	36.1395486	0.2651
0.1	0	0	0.00620507	−0.58441460	0.81143150	0.35552675	35.7639710	0.2813

### Comment

Two other approaches were tried for the co‐axial lens and eye situation: using the (paraxial) second nodal point rather than the ‘finite’ nodal point, and an approach similar to that for the rotating eye situation. Conversions were similar to those given above. Another approach applicable to the co‐axial lens and eye situation was provided by Hastings et al.^43^ for retinal ellipsoids.

## APPENDIX 2 Comparison of MTFs for an initially off‐axis object when viewed peripherally without eye rotation and foveally with a rotated eye

Figures 2 and 3 showed MTFs when a distant object which lies off the common axis of the MS lens and eye is initially viewed peripherally and is then foveally fixated by rotating the eye. These data are replotted in Figure A2. In both cases, the imaging ray bundles pass through lenslet‐covered areas of the MS lens. Strictly, the results are for an object field angle of 32.5 degrees in the peripheral case and 35.8 degrees for the rotated‐eye case; while the field angles differ slightly, this should not greatly affect the comparison.

It is clear that an improved retinal image is obtained following the eye rotation, although this is still inferior to that found when the axes of the MS lens and eye remain coincident and the head is turned to bring the image onto the fovea so that no lenslets lie in the optical path (Figure 1).
